# “You can tell by the way I use my walk.” Predicting the presence of cognitive load with gait measurements

**DOI:** 10.1186/s12938-018-0555-8

**Published:** 2018-09-12

**Authors:** Pritika Dasgupta, Jessie VanSwearingen, Ervin Sejdic

**Affiliations:** 1 Department of Biomedical Informatics, School of Medicine, University of Pittsburgh, Pittsburgh, PA USA; 2 Department of Physical Therapy, School of Health and Rehabilitation Sciences, University of Pittsburgh, Pittsburgh, PA USA; 3 Department of Electrical and Computer Engineering, Swanson School of Engineering, University of Pittsburgh, Pittsburgh, PA USA

**Keywords:** Gait, Cognitive load, Gait classification, Stride extraction, Machine learning, Logistic regression, Support vector machine, Random forest, Learning vector quantization

## Abstract

**Background:**

There is considerable evidence that a person’s gait is affected by cognitive load. Research in this field has implications for understanding the relationship between motor control and neurological conditions in aging and clinical populations. Accordingly, this pilot study evaluates the cognitive load based on gait accelerometry measurements of the walking patterns of ten healthy individuals (18–35 years old).

**Methods:**

Data points were collected using six triaxial accelerometer sensors and treadmill pressure reports. Stride and window extraction methods were used to process these data points and separate into statistical features. A binary classification was created by using logistic regression, support vector machine, random forest, and learning vector quantization to classify cognitive load vs. no cognitive load.

**Results:**

Within and between subjects, a cognitive load was predicted with accuracy values ranged of 0.93–1 by all four models. Various feature selection methods demonstrated that only 2–20 variables could be used to achieve similar levels of accuracies.

**Conclusion:**

Coupling sensors with machine learning algorithms to detect the most minute changes in gait patterns, most of which are too subtle to identify with the human eye, may have a remarkable impact on the potential to detect potential neuromotor illnesses and fall risks. In doing so, we can open a new window to human health and safety prevention.

## Background

A person’s way of walking, or gait, is a congenital human function, but the significance of the way a person walks is often overlooked [1, 2]. Research has shown that the quality of gait can deteriorate with multi-tasking, illness, and age [1, 2]. In fact, the health status of individuals can be recognized by the way they walk [3, 4]. Since gait is heavily dependent on the brain, nerves, and muscles, cognitive tasks performed in concert with walking can change the gait pattern [5]. Thus, for older adults, maintaining balance and stability while walking often requires additional attention [2, 6].

In addition to motor actions, we often think or solve problems [7]. This behavior is an example of dual tasking, which is defined to be the “concurrent performance of two tasks that can be performed independently, measured separately and have distinct goals” [5, 7–9]. Dual-task walking, particularly when a cognitive task is added during walking, can lead to reduced performance in gait quality and can result in cognitive-motor interference (CMI) [5]. CMI results in a subtle cognitive impact upon the gait of healthy and younger adults. Clinically, we can assess the effect of the addition of a secondary cognitive task to the motor activity via reactions to different stimuli (e.g., colors and sounds), manoeuvering through obstacles, using a cell-phone, or counting backward [7].

Cognitive difficulties have been associated with gait changes. For example, disorders of gait have been related to the cognitive problems among those with mood disorders (e.g., depression), dementia-related illnesses (e.g., Parkinson’s and Alzheimer’s diseases), and other motor-cognitive disorders. While age-related cognitive dysfunction such as a decreased ability to appropriately allocate attention may be mild, this additional cognitive load has been related to gait problems. In some of these older adults, the cognitive load has been shown to increase fall risk [7, 9, 10]. For older adults in the United States, falls have devastating consequences and are costly, accounting for a significant number of emergency department visits [11].

However, collection and consolidation of the multiple gait characteristics of human gait can be time-consuming and costly to analyze; these are often captured via wearable sensors such as uni-axial gyroscopes and accelerometers, or other equipment such as pressures sensors, video capture, and other equipment [2, 6, 12]. Many of these instruments generate an immense amount of data per observed person [13, 14]. For instance, captured through accelerometer sensors placed on the chest, back or limbs, gait accelerometry data results in thousands of x–y–z kinematic coordinates through time [15–17]. However, gait analysis via wearable sensors is a popular and inexpensive method, with regard to cost and availability of portable sensors [18]. Wearable sensors are a suitable alternative to larger laboratory equipment, because they still provide many benefits to clinical prognosis, diagnosis, and treatment [18].

Data processing methods such as acceleration signal processing and machine learning can help alleviate the stress of manipulating such large datasets. For example, machine learning has been used with different speeds of walking, foot switches, or other forms of walking combined with clinical outcomes (i.e., Parkinson’s disease) [13, 19, 20]. However, there has been limited research in machine learning for cognitive load classification using gait observations [7–9]. As a first step, it can be particularly useful to do preliminary studies on healthy and young adults in order to establish a basic level of biomechanical movement [21–23]. For example, Mannini et al. state that understanding human physical activity can have future implications in movement technology (including robotics) that could impact the elderly [21]. Thus, differentiating gait qualities due to cognitive load in healthy and young adults can help us prepare a baseline measurement that can be used to assess gait patterns in a more substantial elderly and ill population.

Through the use of machine learning, researchers can harness this data to predict an individual’s gait patterns, cognitive overload, and fall risk [2, 20, 24, 25]. Commonly used algorithms include supervised learning approaches such as logistic regression (LR) [24, 26], support vector machine (SVM) [27, 28], random forest (RF) [29], and k-nearest neighbors (KNN) [30]. However, KNN is negatively affected by increased dimensionality, which is disadvantageous for processing the many features extracted from gait acceleration signal data. An alternative algorithm that bypasses this disadvantage is learning vector quantization (LVQ), a neural network machine learning algorithm, which operates similarly to KNN due to it being a precursor to the nearest neighbor method [31]. There are many studies which have compared SVM with LR, RF, and KNN algorithms; however, there are not many studies that have differentiated between cognitive states using machine learning, particularly with the LR, SVM, RF, and LVQ methods.

The purpose of this study is to demonstrate a range of common machine learning methods that can accurately detect changes in gait with cognitive load in healthy adults. We plan to use a mobile gait data acquisition of stride characteristics of healthy human participants during walking with and without an added cognitive task, and machine learning algorithms, with cross-validation, to describe and classify cognitive status of walking.

## Methods

### Study design and procedures

This study is a prospective cohort study with a repeated-measurements design, where subjects were their own controls. Participation in the study consisted of two sessions in which each session had five stages: affixing the sensors (10–20 min), first walking trial (10 min), a rest period (10–20 min), second walking trial (10 min), and sensor detachment (10 min). The two sessions of this study for each participant occur with at least 48 h between each session. Each session lasted for about 75–90 min.

The walking trials were performed on a treadmill which rested on top of a flat non-compliant surface. The treadmill captured pressure data [in units of Newton force (N)]. The treadmill was set at a steady pace of 2.2 mph, which is 0.98 m/s which is less than the usual adult walking speed of 1.2–1.3 m/s. This treadmill speed was chosen so that all participants would be at a comfortable, and slower than usual walking speed to be closer to a speed common among community-dwelling older adults and persons with walking difficulties. The first walking trial consisted of walking, while the second walking trial consisted of walking while counting backwards from 10,000 in increments of 7, an arithmetic task that is mentally involved [32, 33]. We will refer to the first walking trial as normal walking, while referring to the second walking trial as walking under cognitive load.

**Fig. 1:**
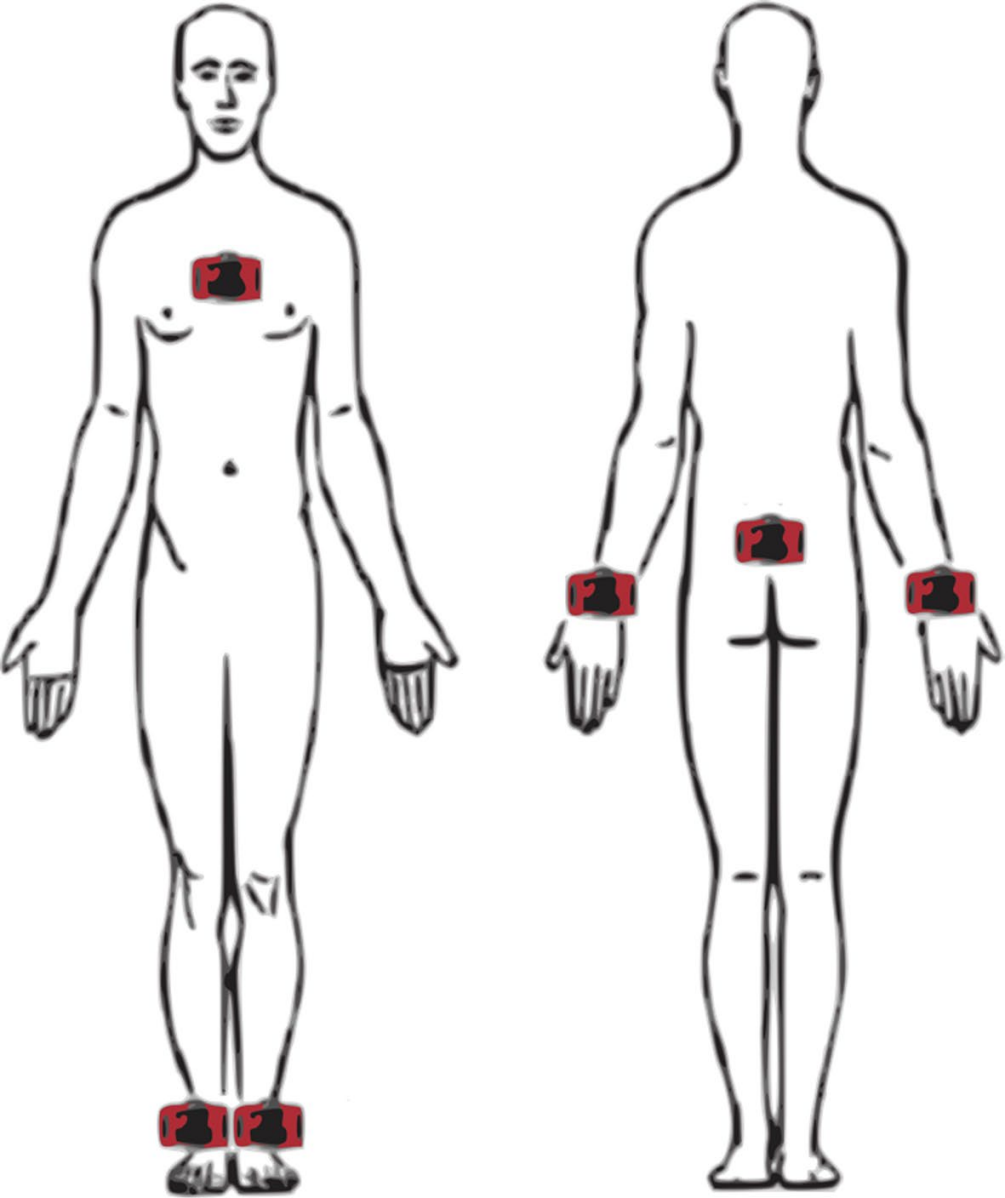
Sensor locations on participants

Participants were affixed with six wGT3X-BT triaxial accelerometer sensors (produced by ActiGraph LLC, Ford Walton Beach, Florida, USA) located on their chest, bilateral ankles, wrists, and lower back (Fig. 1). These sensors captured linear accelerations (in units of $\frac{m}{s^2}$) at a frequency of 80 Hz from the x, y, and z directions which correspond to the mediolateral (ML), vertical (V), and anteroposterior (AP) directions. Overall, each sensor relayed 48,000 data points for each walking trial. The sensors were all clinically accepted monitoring devices and presented minimal risk to the subjects.

All recorded data did not include any personal identifying information. This study’s data collection and analysis was approved by the University of Pittsburgh Institutional Review Board for ethical conduct and participant safety. All data processing and analysis was done in R (versions 3.3.1–3.4.0) [34]. Machine learning methods were implemented using R’s caret package [35].

### Subjects

Volunteers were recruited from the Pittsburgh area in Pennsylvania, USA. Participation in the study was entirely voluntary and could be discontinued at any time. Acceleration and treadmill pressure data were collected from ten healthy volunteers (18–35 years old) at the University of Pittsburgh. Other than age and a subjective perception of healthiness, no other screening criteria were included in the volunteer recruitment. Demographic characteristics, such as gender, height (m), and weight (kg) were taken at the time of the study. Body mass index (BMI) was also calculated (Table 1). Age, gender, and BMI have been known to affect biological outcomes; thus, they are included as covariates in our statistical models.

**Table 1:** Demographic characteristics of study participants

Characteristic	Mean (SD) or N (%)
Age	21.40 (4.38)
Gender = male	5 (50 %)
Height (m)	1.72 (0.09)
Weight (kg)	66.36 (8.41)
BMI	22.87 (1.65)

### Data processing

**Fig. 2:**
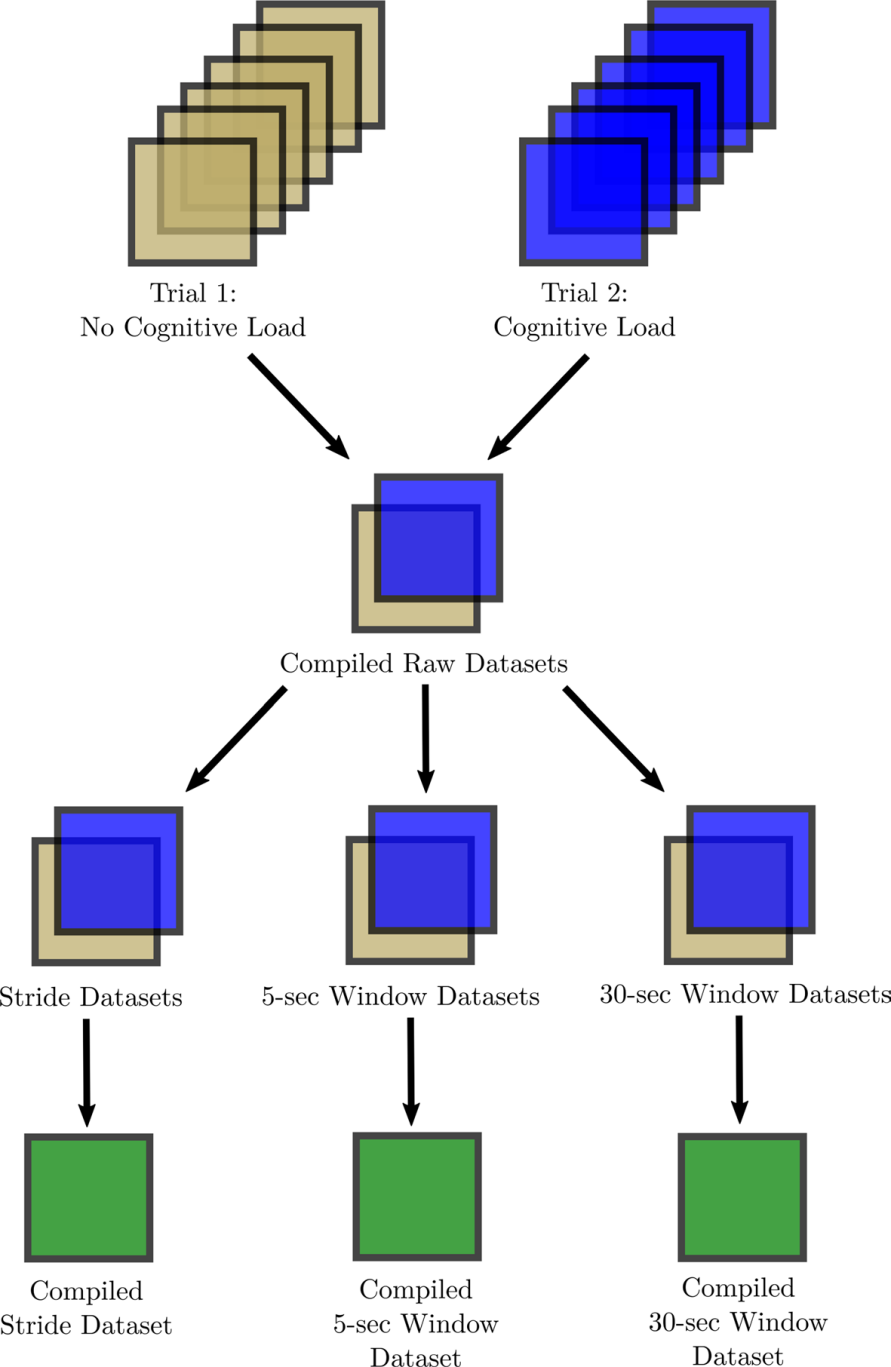
Dataset processing flowchart for each subject

For each subject, a total of 24 raw acceleration datasets were obtained; however, only the 12 raw datasets from session 1 were used due to the participants having a significant difference in cognitive task accuracy, the ratio of number of correct responses (of counting backwards from 10,000 in increments of 7) out of all responses, from session 1 to 2 (paired t-statistic = − 2.94, p-value = 0.017), likely due to a training effect. First, a binary (0/1) variable called “cognitive load” was created to represent each trial, where “cognitive load” would be set to 1 (trial 2) and 0 otherwise (trial 1). Second, for each trial, the 6 raw datasets were compiled into one compiled raw dataset. Third, three data transformations were done on these datasets to obtain a stride dataset and two observation window datasets. Fourth, for each transformation, the datasets for trial 1 and trial 2 were combined. This resulted in a total of 30 datasets (3 processed datasets per subject in session 1) (Fig. 2).

#### Stride extraction

Acceleration signal based stride extraction is a clinically useful tool to evaluate cognitive changes while walking concerning the gait cycle [36]. Successive heel strikes and toe-offs on the same side have been used to define stride, stance, and swing intervals [37]. Heel strike and toe-off events were extracted from the lower back sensor using the algorithm outlined in Sejdić et al. [38]. Local minima points in the V direction correspond to toe-offs, and local minima points in the AP direction are related to heel strikes [38]. The order of which foot initially made the first step was found by taking the average of the first 10 ms of acceleration in the ML direction [38]. If there was positive mean value, the right foot came first; otherwise, the left foot came first [38].

A condensed description of this algorithm consists of the following steps: (1) pre-processing gait accelerometry signals via median filters, (2) determine which foot came first by calculating the average of the mediolateral acceleration signals in the first 10 ms, (3) capture heel strike events via the local minima points in the AP signals, and (4) capture toe-off events via the local minima points in the V signals.

**Fig. 3:**
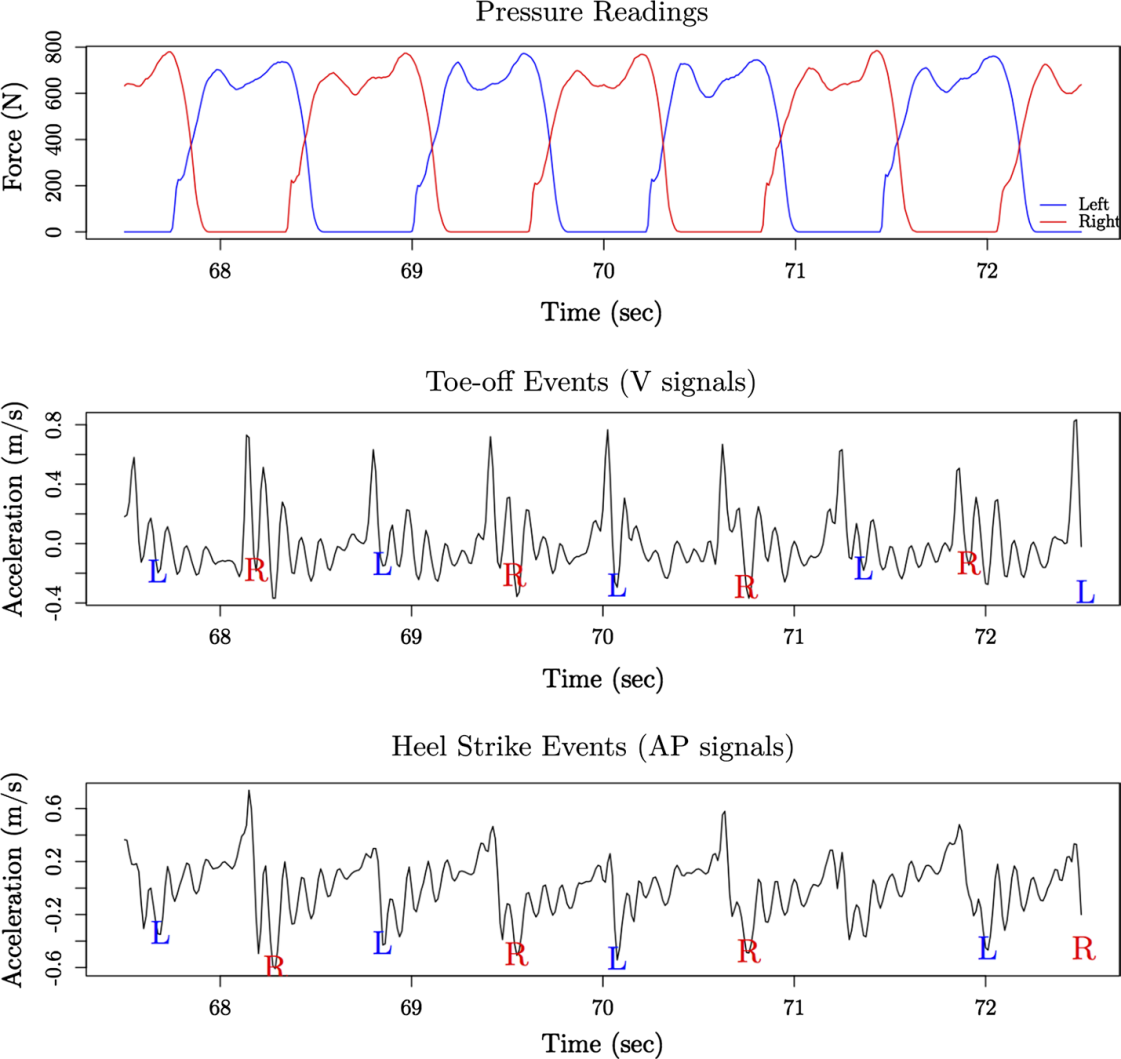
Stride extraction sample. Subject 1’s toe-offs and heel-strikes, which were determined from foot pressure recordings during treadmill walking and acceleration signal recorded using the lower back sensor. The top graph shows the pressure readings from the treadmill, the middle graph are the V acceleration readings, and the bottom graph are the AP acceleration readings. Red and blue colored lines and labels depict the right and left foot respectively

Foot pressure based treadmill reports were used to validate sensor-derived heel strike and toe-off events. When the subject pushes their foot off the ground, the pressure reduces to 0 N; conversely, when the subject heel strikes, the pressure increases. Using a technique from Truong et al. [39], an on/off filter was created to detect heel strike and toe-off events for each foot. This on/off filter produced time points for when the foot was on or off the ground. These time points were matched up to the stride events for validation of Sejdić et al.’s method. An example of stride extraction and validation is shown in Fig. 3.

#### Window extraction

**Fig. 4:**
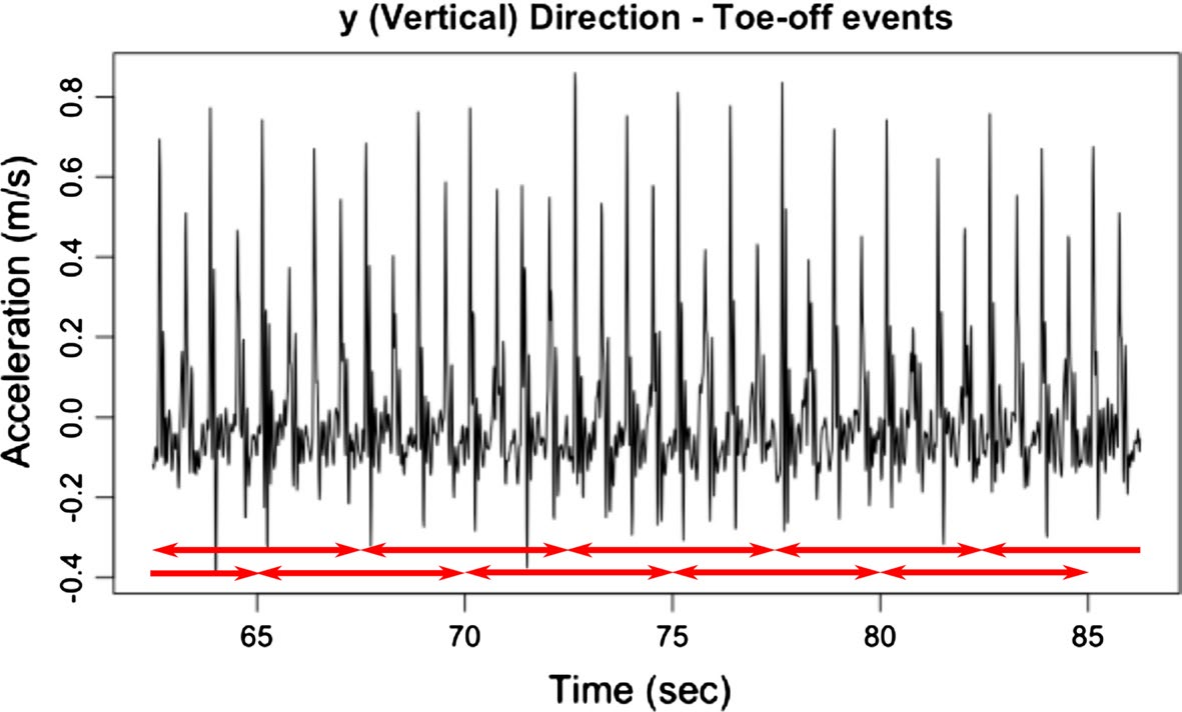
Acceleration signal extraction during walking. Subject 3’s Vertical acceleration signals from the back sensor. 5 s observation windows with 50% overlap are shown

A more straightforward alternative to stride extraction is the creation of sliding observation windows, by partitioning sensor signals into smaller time segments [40]. Partitioning into sliding observation windows is a conventional technique in activity monitoring and machine learning analysis of accelerometer data [21, 40]. For each subject and each sensor, two datasets were formed: (1) acceleration signals were split up in 5-s intervals with 50% overlap, and (2) acceleration signals were split up in 30-s intervals with 50% overlap. An example of how window extraction is done is shown in Fig. 4.

#### Errant data removal

Data points were removed to eliminate any start-up, pausing, and ending effects to allow the subject to become familiar with getting on and off the treadmill. For the stride datasets, the first fifty strides and the last five strides were removed. Outliers based on average stride time were removed using the interquartile range (IQR) rule [41]. Comparing the remaining strides is a statistical challenge because strides are characterized by vectors of unequal lengths, and there are hundreds of strides to compare.

Thus, an ANOVA test [null hypothesis: no statistical difference in means of stride features (see below in “Feature extraction” section) between strides] was done before and after errant data removal and resulted in a p-value of 2.2e−16 and 1, respectively. These results indicate that errant data removal was done correctly because the processed strides were not distinguishable from the other. For the observation window datasets, the first and last minute of the data were removed.

#### Stride time differences

Additionally, within each participant’s stride extracted data (n = 1351 to 1870 strides), an ANOVA test was done between the stride time distributions between the trials. The null hypothesis of this ANOVA test was “no statistical difference in the means of stride times between strides”. Typically, a large sample size will lead to statistical significance; in this case, the stride times are expected to be significantly different.

### Features

#### Feature extraction

In order to capture descriptors from each stride and window, it is common in gait studies [24, 42] to calculate descriptive statistics from strides and windows. For each direction (V, AP, and ML), twelve features were extracted from each stride and each observation window in each of the six sensors. These twelve features were the mean, standard deviation, pair-wise correlation, and pair-wise covariances of each of the three directions [43]. From the stride data specifically, times and lengths of the stance phase, swing phase, and overall stride were also extracted. As a result, a 78-feature vector described each stride whereas a 72-feature vector described each observation window.

#### Feature selection

High dimensionality may lead to over-fitting in machine learning analysis, and it is advantageous to reduce the number of features. First, each of the four models was run with all the features. Second, in order to select which features to include in machine learning models, the following methods were performed: (1) for all four models, a correlation matrix between each feature was constructed; highly correlated feature pairs (r > 0.75) were found and within each pair, the feature with the highest mean absolute correlation was removed; (2) the LR model ran step-wise variable selection, via a likelihood ratio test, which selects the model with the lowest Akaike information criterion (AIC) value [44]; (3) in the LVQ model, features were ranked by the absolute value of the t-statistic for each feature parameter; and (4) in the RF and SVM models, recursive feature elimination was done [45]. Some feature selection methods were utilized so that decreased time for feature reduction and a consensus of essential features could be reached for each model.

### Machine learning models and evaluation

The presence of a cognitive load, represented by a binary (0/1) variable, was classified based on gait feature vectors. For binary outcomes, the following four machine learning algorithms were used: LR, LVQ, SVM, and RF. Well-known evaluation metrics were used such as accuracy, sensitivity, specificity, and area under the curve (AUC). In this report, we present the accuracy (Eq. 1) values, with their 95% confidence intervals; sensitivity, specificity, and AUC values are reported in Appendix (Figs. 8,  9,  10,  11,  12).

1 $$\begin{aligned} Accuracy = \frac{TP+TN}{TP+TN+FP+FN} \end{aligned}$$

where TP is the number of true positives, i.e., the model identifies a cognitively loaded stride/window that was labelled as cognitively loaded; TN is the number of true negatives, i.e., the model identifies a non-cognitively loaded stride/window that was labeled as not cognitively loaded; FP is false “cognitive” load identifications; and FN is false “no cognitive load” identifications.

For each of the machine learning algorithms, we employ three different modelling strategies: within subjects, between subjects, and leave one out. Feature selection was performed after the datasets were processed for each model.

#### Within and between subjects

For the within-subjects model, each subject’s dataset was combined within each of the three data types: strides (n = 1351 to 1870 strides), 5-s observation windows (n = 1280 windows), and 30-s windows (n = 320 windows). For the between subjects model, all subjects’ datasets were combined within each of three data types: stride (n = 16,291 strides), 5-s observation windows (n = 12,800 windows), and 30-s windows (n = 3200 windows).

Datasets were split into training and test datasets; datasets were randomly split, using a seed of 7 and the sample function in R, into 80%, which was used for training each machine learning model, and 20%, which was used to evaluate the model’s performance. The tenfold cross-validation method was used on each of the training sets, where the training dataset was split into ten subsets. Each subset was held out while the model was trained on all other subsets. This cross-validation process was repeated three times. The final model was chosen by the best accuracy of these runs. Then this model is run on the test dataset to validate the model.

#### Leave one subject out validation

A leave one subject out model was done to truly capture how well other subjects’ features and data can predict an individual’s cognitive load. This was done by combining all subjects’ datasets for only the stride data type. Each model was trained on nine out of the ten subjects (n = 14,421 to 14,940 strides), and the model was tested on the remaining subject (n = 1351 to 1870 strides). Training and testing were done ten times, and average evaluation metrics were calculated.

## Results

An equal number of adult males and females participated. The participants reported good health, which was consistent with the mean BMI derived in the good condition range (Table 1). All participants completed both study sessions.

### Stride characteristics

Within each of the ten subjects, the stride time distributions were not significantly different between trials using ANOVA tests with a Tukey’s posthoc test (Table 2). Earlier, we indicated that we expected the stride times to be significantly different due to a large sample size of strides per subject. However, the test indicated differences in stride times cannot be differentiated, which suggests that machine learning algorithms will have to be very sensitive when differentiating between cognitive load vs. no cognitive load.

**Table 2:** Number of strides and stride time comparison between trials 1 and 2, for each subject

Subject	Stride no.	Stride time (s) [mean (SD)]	
		No cognitive load	Cognitive load
1	1511	1.32 (0.23)	1.29 (0.16)
2	1688	1.17 (0.12)	1.20 (0.04)
3	1592	1.28 (0.18)	1.29 (0.07)
4	1705	1.22 (0.17)	1.22 (0.07)
5	1810	1.13 (0.10)	1.12 (0.06)
6	1721	1.18 (0.12)	1.20 (0.09)
7	1870	1.10 (0.11)	1.11 (0.08)
8	1565	1.22 (0.07)	1.25 (0.03)
9	1478	1.24 (0.16)	1.24 (0.04)
10	1351	1.24 (0.07)	1.31 (0.09)

### Machine learning results

For each model, all machine learning results reported were derived from the results of the test dataset.

**Fig. 5:**
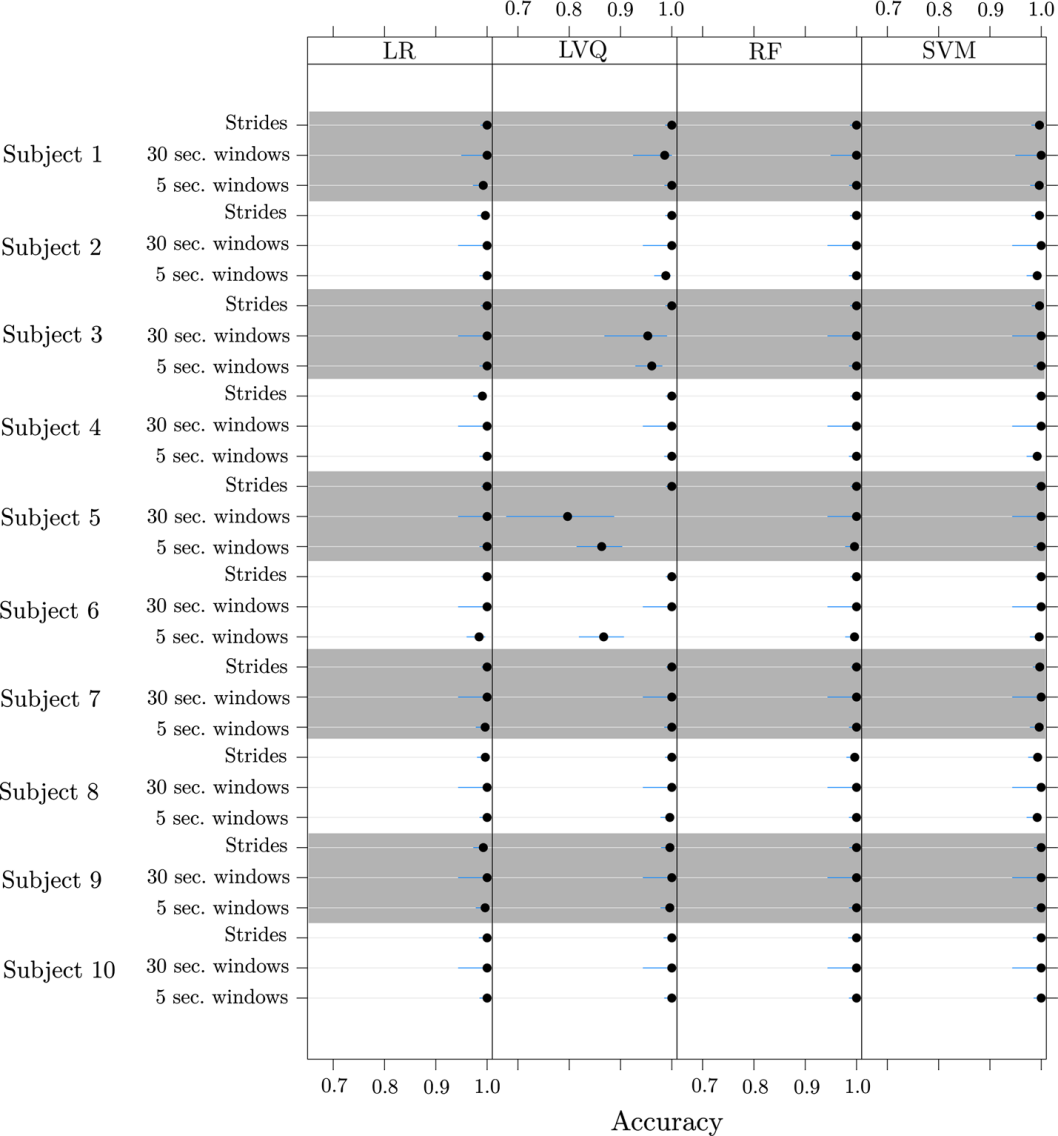
Within subjects results. Accuracy values (95% confidence intervals in blue) using the “within subjects” model using all three dataset types

Within each subject, Fig. 5 depicts the accuracy values from all the models. From the strides datasets, over all subjects, the mean (95 % t-distributed CI) of the accuracy of LR is 0.998 (0.995, 1.00), LVQ is 0.999 (0.999, 1.00), RF is 0.998 (0.999, 1.00), and SVM is 0.998 (0.996, 0.999). From the 30 s. window datasets, over all subjects, the mean (95% t-distributed CI) of the accuracy of LR is 1.0 (1.0, 1.0), LVQ is 0.97 (0.93, 1.0), RF is 1.0 (1.0, 1.0), and SVM is 1.0 (1.0, 1.0). From the 5 s. window datasets, over all subjects, the mean (95% t-distributed CI) of the accuracy of LR is 0.997 (0.993, 1.00), LVQ is 0.97 (0.93, 1.0), RF is 0.999 (0.998, 1.00), and SVM is 0.996 (0.994, 0.998).

Common influential features that appear for most participants in these models are the means and standard deviations of the x, y, and z directions for the back sensor and ankle sensors.

**Fig. 6:**
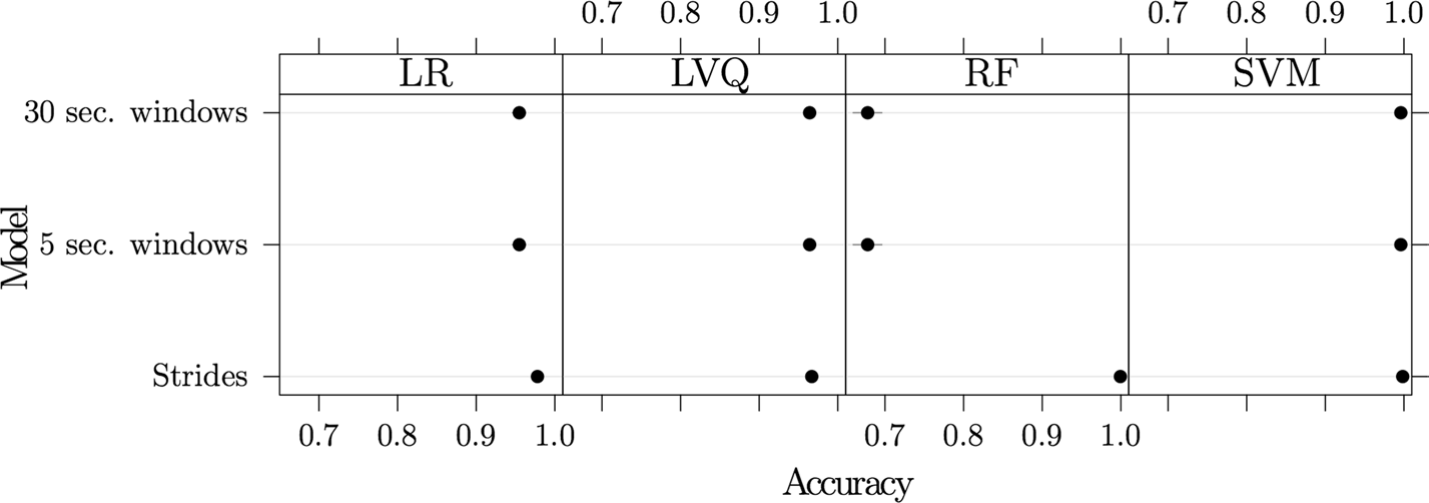
Between subjects results. Accuracy values (95% confidence intervals in blue) using the “between subjects” model using all three dataset types

Between all the subjects, Fig. 6 depicts the accuracy values from all the models. Common influential features that appear for most participants in these models were similar to the within-subjects models, with the inclusion of the covariances and correlations of the x, y, and z-direction of the chest sensor.

**Fig. 7:**
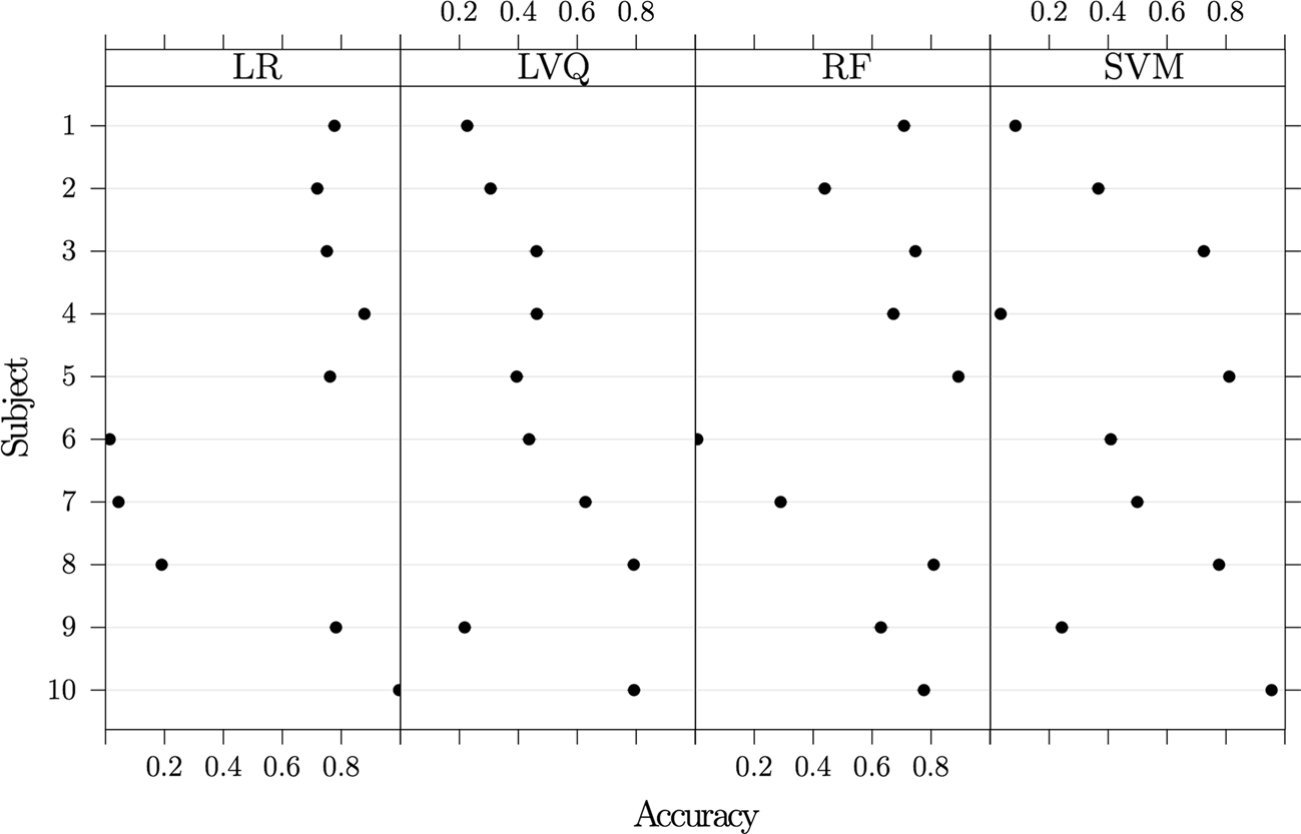
Leave one subject out results. Accuracy values (95% confidence intervals in blue) using the “leave one subject out” model

By training on nine out of ten subjects and testing on the remaining one, Fig. 7 depicts the accuracy values from all the models for each tested subject. Over all subjects, the mean (95% t-distributed CI) of the accuracy of LR is 0.59 (0.33, 0.85), LVQ is 0.47 (0.32, 0.62), RF is 0.60 (0.40, 0.79), and SVM is 0.49 (0.26, 0.72). Feature reduction varied greatly for these models, likely due to the inconsistency of accuracy results from one subject to the other.

**Fig. 8:**
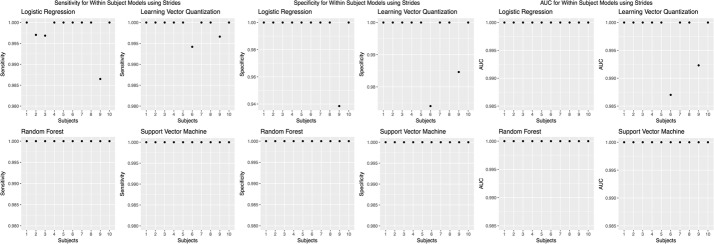
Sensitivity, specificity, and AUC values for the within subjects (stride dataset) model. Sensitivity values using the “within subjects” model using the stride dataset

**Fig. 9:**
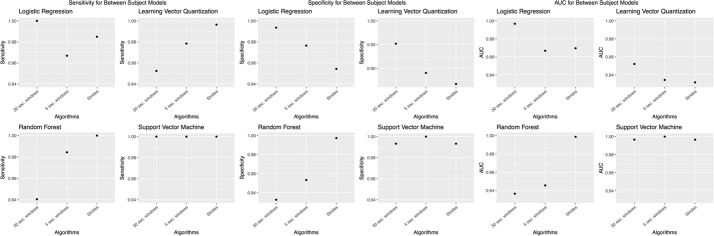
Sensitivity, specificity, and AUC for the between subjects model. Sensitivity values using the “between subjects” model

**Fig. 10:**
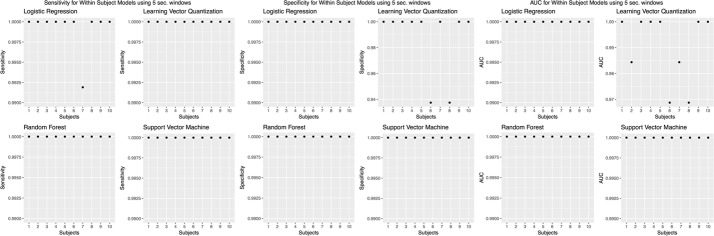
Sensitivity, specificity, and AUC for the within subjects (5 s windows) model. Sensitivity values using the “within subjects” model using 5 s windows

**Fig. 11:**
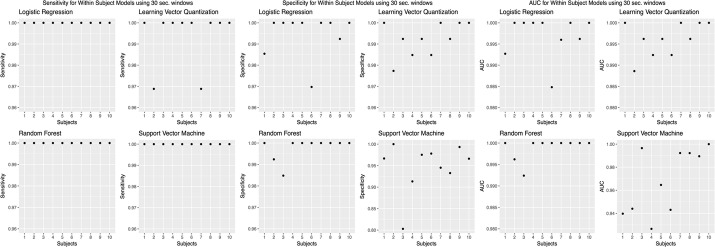
Sensitivity, specificity, and AUC values for the within subjects (30 s windows) model. Sensitivity values using the “within subjects” model using 30 s windows

**Fig. 12:**
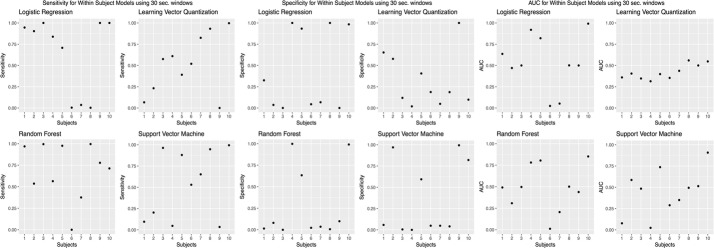
Sensitivity, specificity, and AUC values for the leave one subject out model. Sensitivity values using the “leave one subject out” model using the stride dataset

We chose to present only accuracies, which is measured by dividing the number of correct predictions by the number of predictions [46]. However, accuracy was not the only evaluation metric; we calculated other evaluation metrics, such as sensitivity, specificity, and AUC values (Figs. 8,  9,  10,  11,  12).

## Discussion

This study’s purpose was to implement signal processing on raw accelerometry gait data and evaluate the performance of common machine learning methods, LR, RF, LVQ, and SVM, to classify the presence of cognitive load in ten healthy adults.

Our machine learning models consisted of within-subject, between-subject, and leave one subject out classification. Within-subject classification is clinically vital for precision (individual-specific) medicine because it can help health-care practitioners and researchers more accurately predict which treatment strategy will work for a particular person, without regard for differences in individuals [47, 48]. Leave one subject out classification is also relevant for precision medicine, and our attempt with ten individuals is an example of how other people’s gait patterns can be used to train a machine learning model to test on an individual. For example, model training on similar patients can be done to help decide a treatment strategy for a patient. Conversely, between-subject classification only assesses an overall baseline of gait patterns over a population.

Not all four machine learning algorithms performed with consistently high accuracy, among all three modelling approaches. In the within subjects model, The results show that the LR, RF, and SVM algorithms recognized cognitive load accurately (with accuracy > 0.93) for both strides and windows. In particular, RF and SVM were consistently strong performers amongst all data transformations, whereas LVQ performance varied. As seen in Fig. 5, subjects 3, 5, and 6 had varied LVQ accuracy values for window datasets. Upon further inspection, feature selection with LVQ for the window datasets was not able to reduce the number of features. Feature selection was able to reduce the number of features in LR, RF, and SVM, but not in LVQ which could mean that there are too many features in the model, which in turn could be picking up random noise instead of the actual trend leading to erratic results. In the between subjects model, LR and SVM algorithms performed well, and LVQ and RF were weak performers. LVQ being a weak performer for both models is a surprising result because LVQ has proven to be a reliable gait predictor in the past [49].

When we assessed the leave one subject out approach, many of the accuracy values were lower than random chance. It was clear that due to gait differences between the individuals, cognitive load prediction varied considerably. Even though the data points for this approach were from the stride dataset and the sample size of these datasets were large when put together for the training set, the result of the machine learning algorithms suffered from having a limited overall sample size of ten individuals. Each individual, albeit being similar in basic demographic characteristics, presumably have different gait patterns. A low accuracy on the leave on out approach in this study may require the following in order to get better results: (1) a high sample size of participants, (2) more detailed demographic and medical characteristics of each participant, (3) different signal processing techniques, and/or (4) varied feature selection approaches by either choosing different features or changing the feature reduction technique. Since this approach has precision medicine implications and can be a bridge between the results of the between subjects approach and the within subjects approach, it is relevant to our discussion. We hope that with a higher sample size, the leave one subject out approach will produce higher accuracy results; however these results do not discount the high accuracy results of the within subjects approach.

Moreover, in machine learning, overfitting can become an issue, when there is low training error and high generalization error. In particular, overfitting can occur when there is a higher model complexity (too many parameters) or a small sample size. To overcome overfitting in our models, we performed feature selection. Feature selection resulted in 2–20 features per model used. Common features included ML signals in back acceleration, stride time and length, V signals in chest acceleration, all signals in both wrist acceleration, and all signals in both ankle acceleration. This suggests that collecting data from one sensor, such as the back, chest, wrists, or ankles, will be sufficient to determine differences in cognitive load. We also evaluated overfitting by including sensitivity, specificity, and AUC values in our Appendix. Specificity and sensitivity values were consistently above 0.90 for all algorithms and for the within and between subjects models.

Overall, these findings are particularly compelling because the treadmill is a “dedicated pacer” and the subjects’ were very similar to each other in both demographic and gait characteristics, meaning that the machine learning algorithms, particularly in the within subjects model, were sensitive enough to pick up the cognitive load.

Statistical signal processing is a time-consuming task, but stride extraction was performed successfully and validated by the treadmill pressure reports for each subject (Fig. 3). Even though stride extraction was done for each subject, window extraction was also done to compare how predictive machine learning algorithms can be without respect to gait cycle events. In the within subjects model, accuracy values between windows and strides were easily comparable, with the exception of the LVQ results. Due to the time and memory consuming task of signal processing, window extraction is preferable [50].

The data collection and analysis process had multiple limitations. First, the use of the treadmill is not reflective of overground walking, which could lead to poor generalizability in using this technique and these models for further gait analysis. However, after treadmill familiarization, young and unimpaired individuals, particularly healthy 18–28 year olds, have been shown to have negligible differences or no differences at all between overground and treadmill gait parameters and leg kinematics [51–53]. Second, the low subject sample size (n = 10) may have led to poor accuracy values for the leave one subject out method. However, the stride/window data used for the leave one subject out method had sample sizes that were sufficiently large (please see “Leave one subject out validation” section). Even though there may be low generalizability of the gait data acquired by these subjects, future work in this area has the potential to be remedied by a higher subject sample size and a more extensive set of acceleration signals. Third, the cognitive task accuracy was not included in the machine learning models. Cognitive task accuracy or other measures of how much cognitive load a person had during the walking tasks could have biased the accelerometer data. For example, an individual could have had more cognitive loading than another individual. However, this issue is partially alleviated due to the fact that the machine learning algorithms classified between the presence of cognitive load versus no cognitive load. We can see that this bias could have possibly contributed to the poor leave one subject out results.

These findings contribute to the era of personalized medicine, which aims to improve the potency of therapies at an individual level. To provide personalized medicine in gait research, we must identify appropriate gait features that reliably predict differences in cognitive load. Sensors are relatively inexpensive, and they can be used by healthcare professionals and patients alike to track gait patterns. However, the analysis of raw sensor data can be challenging without an appropriate tool; window separation of these acceleration signals and machine learning analysis fills this gap. The implications of this analysis can lead to the creation of a medical device that can be used in not only the clinic but also in athletics.

Marrying sensors with machine learning algorithms has the potential to be an early indicator of disease or fall risk, especially in older adults. Ageing has been known to contribute to gait difficulties; even more so, gait impairment may be due to an intrinsic disease [54]. In fact, those with gait disorders are more likely to have dementia symptoms than those without gait disorders. We can endeavour to identify these pre-clinical changes in gait that are associated with a cognitive load for diagnosis or aid in other therapies, such as combative cocktail therapies for neuromotor illnesses [55, 56].

Also, there is a quiescent need to identify cognitive status via different types of gait disorders. While this study discriminated between two types of cognitive statuses, this analysis has the potential to describe the overall spectrum of cognitive disorders. Granted, this analysis will heavily depend on the population being studied, but separating the several neurological causes for gait disorders, such as stroke, ataxia, and Parkinson’s disease [54], can help add to the stock of knowledge of gait and cognitive faculties.

Thus far, we have determined cognitive status with previously collected gait data. Future work consists of predicting forthcoming continuous clinical outcomes from gait data, predicting the acceleration signals of the next stride using forecasting algorithms such as hidden Markov models and autoregressive integrated moving average, along with other gait parameters, such as cadence.

## Conclusion

In our study, we found that just by combining machine learning technology with advanced signal processing methods on sensor data, we were able to detect cognitive states accurately. Our features were derived from gait accelerometry signals from healthy adult subjects. Moreover, we determined that using window extraction methods and selecting gait features from data from only one sensor is satisfactory. These results have an array of clinical implications, namely in personalized medicine and early detection of neuromotor diseases. This successful pilot study provides a clear path for expansion, due to its explicit findings and its potential application towards a much larger population consisting of adults across a broad range of ages. Lastly, this is particularly provocative due to the fusion of machine learning on gait accelerometry data that could lead to the prediction of disease or gait instability in older adults.

## Appendix

See Figures 8,  9,  10,  11,  and 12.
